# Entwicklung, Ausmaß und Determinanten der psychischen und emotionalen Erschöpfung bei Erzieherinnen und Erziehern

**DOI:** 10.1007/s40664-022-00468-8

**Published:** 2022-05-31

**Authors:** Nadine Madeira Firmino, Jürgen Bauknecht

**Affiliations:** 1 Fliedner Fachhochschule Düsseldorf, Düsseldorf, Deutschland; 2 Hochschule Koblenz, Koblenz, Deutschland

**Keywords:** Belastungsfaktoren, Resilienzfaktoren, Berufsgruppenvergleich, Kindergarten, Frühkindliche Bildung, Stress factors, Resilience factors, Professional group comparison, Kindergarten, Early childhood education

## Abstract

Der vorliegende Beitrag untersucht die psychische Erschöpfung bei Erziehern und Erzieherinnen in den Jahren 2006, 2012 und 2018 und die emotionale Erschöpfung in den Jahren 2012 und 2018 jeweils im Vergleich zu den Angehörigen anderer Berufsgruppen (Datenbasis: „Erwerbstätigenbefragungen“ des Bundesinstituts für Berufsbildung [BIBB] und der Bundesanstalt für Arbeitsschutz und Arbeitsmedizin [BAuA]). Innerhalb der beiden Gruppen (Erzieherinnen und Erzieher/andere Berufe) werden ebenso Untergruppen nach Alter und Geschlecht untersucht. Gemäß ihrer psychischen und emotionalen Erschöpfung werden die Erzieherinnen und Erzieher in vier Gruppen unterteilt. Zunächst wird die Verteilung verschiedener Belastungs- und Unterstützungsfaktoren bei pädagogischen Fachkräften einerseits sowie bei den Angehörigen anderer Berufe andererseits dargestellt. Im Anschluss wird ermittelt, wie diese Faktoren die Wahrscheinlichkeit der Zugehörigkeit zur besonders stark belasteten Gruppe beeinflussen. Schließlich wird dargelegt, welche Belastungsfaktoren reduziert werden können, um die psychische und emotionale Erschöpfung bei Erzieherinnen und Erziehern zu reduzieren. Es kristallisieren sich einzelne Belastungsfaktoren heraus, bei denen aufgrund hoher Werte bei Erzieherinnen und Erziehern Spielraum für Verbesserungen vorhanden ist und die gleichzeitig bedeutsam für die Erschöpfung sind.

## Abstract (en)

This paper investigates mental exhaustion of educators in the years 2006, 2012 and 2018 as well as emotional exhaustion for the years 2012 and 2018, in both respects in comparison to other educational groups (data base: “Employee Surveys” carried out by the Federal Institute for Vocational Education and Training [BIBB], and the Federal Institute for Occupational Safety and Health [BAuA]). Intragroup comparisons of educators as well as other occupational groups show differences according to age and sex. Based on their mental and emotional exhaustion, educators are categorized into four groups. Then the prevalence of different stress factors amongst educators and other occupational groups is shown. It is determined how these stress factors affect the probability to belong to the group with the highest mental and emotional exhaustion. Finally, it is shown which stress factors can be reduced in order to reduce educators’ mental and emotional exhaustion. There are single stress factors with leeway for improvements due to high levels amongst educators and which, at the same time, are crucial for exhaustion.

Zahlreiche Befunde belegen, dass Erzieherinnen und Erzieher unter psychischer und emotionaler Erschöpfung leiden [1, 5, 9, 10, 14, 16, 21, 22]. Das Aufgabenspektrum dieser Berufsgruppe reicht von der Umsetzung des Bildungsauftrags und konstruktiver Zusammenarbeit mit Eltern über Kinderschutz bis zur Integration von Kindern mit Fluchterfahrung. Zudem führt der aktuelle Fachkräftemangel zu hoher Belastung. Pädagogische Fachkräfte sind vermehrt Stressfaktoren (wie Lärm, zusätzlicher Verantwortung, Zeitdruck) ausgesetzt [15, 21].

Zwar erscheint es evident, dass pädagogischen Fachkräfte ein erhöhtes Risiko für psychische Belastungen tragen [5, 10]. Unklar bliebt jedoch in komparativer Perspektive, welche Belastungsfaktoren für die psychische und emotionale Erschöpfung ausschlaggebend und wie stark diese ausgeprägt sind. Der Beitrag identifiziert Bereiche, in denen Spielraum für Verbesserungen besteht und gleichzeitig eine Verringerung dieser Belastungsfaktoren die Erschöpfung reduzieren kann. Zeitgleich werden Belastungsfaktoren identifiziert, die bei Erzieherinnen und Erziehern im Vergleich zu den Angehörigen anderer Berufsgruppen nicht stark ausgeprägt sind und/oder die für die Erschöpfung kaum relevant sind. Hierfür werden Daten der „BIBB/BAuA-Erwerbstätigenbefragungen – Arbeit und Beruf im Wandel, Erwerb und Verwertung beruflicher Qualifikationen“ aus den Jahren 2006, 2012 und 2018 analysiert.

## Theoretischer Hintergrund

Pädagogische Berufsarbeit stellt hohe Anforderungen an Fachkräfte. Im arbeitspsychologischen Stressmodell spielen individuelle Bewertungen arbeitsplatzspezifischer Bedingungen eine ausschlaggebende Rolle für das Stresserleben. Negative Belastungsfolgen resultieren aus einer unpassenden Bewältigung von Anforderungen innerer sowie äußerer Faktoren. Thinschmidt und Stück [19] unterscheiden dabei zwischen folgenden kurzfristigen Beanspruchungsfolgen:

- Ermüdung (durch Überforderung),
- Monotonie (durch Unterforderung),
- psychische Sättigung (durch Frustration),
- Stress (durch Bedrohung, Diskrepanz Individuum ↔ Umwelt).

Wird den Belastungsfaktoren aus internen (Resilienz) oder externen (z. B. Unterstützung durch den Träger) Quellen nichts entgegengestellt, können langfristige Beanspruchungsfolgen entstehen, welche sich in dauerhafter psychischer und emotionaler Erschöpfung zeigen.

Die psychische Belastung wird durch die steigende Komplexität der Arbeitsanforderungen begünstigt. Eine Besonderheit des Berufsfelds besteht zudem in der hohen Personalfluktuation, den steigenden Anforderungsprofilen, der Digitalisierung, der Flexibilisierung von Arbeitszeit und der niedrigen Entlohnung. Dies kann bei der Berufsgruppe zu Stress und psychischer Erschöpfung führen [19]. Der zunehmende Leistungsdruck und die steigenden Erwartungen an ein sich stets wandelndes Berufsprofil führen somit zu einem Anstieg an empfundenen Belastungen [13–21].

Die vorliegende Analyse orientiert sich am arbeitspsychologischen Stressmodell [4, 22]. Dieses berücksichtigt personenbezogene (eigene Merkmale wie z. B. Alter, Vorerkrankungen, Motivation) und bedingungsbezogene (externe Merkmale wie z. B. Rahmenbedingungen des Arbeitsumfelds) Stressoren bzw. Ressourcen, deren Bewältigung oder Nichtbewältigung und die daraus resultierenden Folgen. Gemäß diesem Modell sind individuelle Bewertungen arbeitsplatzspezifischer Bedingungen relevant für das Stresserleben. Die bedeutsamsten Ressourcen sind eigene Handlungs- und Entscheidungsspielräume bei der täglichen Arbeitsgestaltung durch den Träger [8] sowie die soziale Unterstützung durch die Kolleginnen und Kollegen. Personenbezogene Ressourcen wie Motivation, fachliche Kompetenzen und Reflexionsfähigkeit tragen zur Bewältigung von herausfordernden Situationen bei [4]. Die Arbeitsbedingungen in Kindertageseinrichtungen können schematisch in Anlehnung an Thinschmidt [20] in die folgenden Bereiche unterteilt werden (Tab. 1):

**Table 1:** 

Arbeitsumgebung	Lärm Luftbeschaffenheit Räumliche Bedingungen Vorhandene Mittel (Ausstattung)
Organisatorische Bedingungen	Art und Größe der Kita Pädagogisches Konzept der Kita Gruppengröße Gruppenzusammensetzung Anzahl der Mitarbeiter im Team Arbeitszeit (Arbeitszeitumfang, Schichtarbeit) Pausenregelung
Anforderungen aus der Arbeitsaufgabe	Aufgabeninhalte (Komplexität, Vielfalt, Sinnhaftigkeit, Kontrollmöglichkeiten) Informationsdichte (Anzahl parallel auszuführender Aufgaben) Zeitdruck (Vor- und Nachbereitungszeiten) Daueraufmerksamkeit (Beaufsichtigung der Kinder) Verantwortlichkeit (für die Gesundheit der Kinder) Physische Belastung (Heben und Tragen der Kinder, ungünstige Körperhaltungen) Sprech- und Stimmbelastung Emotionale Anforderungen (Diskrepanz zwischen äußerer und innerer Gefühlslage) Berufliche Entwicklung (Fort- und Weiterbildungen) Gefahrquellen (Treppen, Nässe, Spielzeug und Ausstattung)
Soziale Bedingungen	Sozialstruktur der Klientel Teammerkmale (Altersstruktur, Qualifikationsniveau, Rolle) Betriebsklima Führung
Gesellschaftliche Bedingungen	Wirtschaftliche Lage Berufsstatus und -image Bezahlung

Anforderungsverursachte Belastungen können einerseits positiv empfunden werden, andererseits Gesundheit, Wohlbefinden und Arbeitszufriedenheit reduzieren. Laut Thinschmidt [20] sind für das Ausmaß von Belastungsfolgen die Person selbst mit ihren Bewältigungsstrategien, die sozialen und organisationalen Ressourcen der Einrichtungen als auch die gesellschaftlichen Bedingungen verantwortlich.

## Forschungsstand

Erzieherinnen und Erzieher erleben sich im Spannungsfeld zwischen ungenügenden strukturellen Rahmenbedingungen und steigendem Aufgabenspektrum, welches wiederum in verschiedenen Belastungsformen, wie etwa psychischen und emotionalen Erschöpfungsmodi mündet. Die von Thinschmidt [19] aufgelisteten branchentypischen Belastungsfaktoren (s. Tab. 1) konnten in verschiedenen Untersuchungen belegt werden [1, 2, 7, 9–20].

So zeigen die Ergebnisse der GEW-Studie (*n* = 947) von Rudow [14] bereits 2004, dass „neben den körperlichen, die psychischen Belastungen und ihre Auswirkungen auf die Leistungsfähigkeit und Gesundheit ein Hauptproblem in der Arbeit von Erzieherinnen“ (S. 2) darstellen. Als Ursachen werden in der baden-württembergischen Untersuchung Lärm, die Diversität an Arbeitsaufgaben, Zeitdruck als auch strukturelle Herausforderungen (z. B. Personalmangel, unzureichende Unterstützung seitens des Trägers) von den Befragten genannt.

Kliche et al. [10] schlussfolgern auf Grundlage ihrer 2005–2006 durchgeführten Erhebung zu Prävention und Gesundheitsförderung in Kitas u. a., dass Erzieherinnen und Erzieher vielfach von Infektionserkrankungen, Einschränkungen des Bewegungsapparats sowie emotionaler Erschöpfung betroffen sind. Zu ähnlichen Erkenntnissen kommt eine qualitative Befragung in Bremen. Die Ergebnisse zeigen, dass es neben ausgeprägten physischen auch deutliche psychische Belastungen bei der befragten Berufsgruppe gibt. Diese variieren jedoch nach Sozialraum und Schwerpunktgruppen [1].

Jungbauer und Ehlen [9] zeigen in ihrer Querschnittuntersuchung (*n* = 773), dass über ein Drittel der befragten Fachkräfte von klinisch relevanten psychischen und psychosomatischen Beschwerden betroffen ist, welche vermutlich durch Stress im Job bedingt sind. Die Erkenntnisse der Analyse der Techniker Krankenkasse zeigen zudem, dass überwiegend psychische Erkrankungen der Grund für Krankschreibungen sind. Im Vergleich zum Bundesdurchschnitt kamen pädagogische Fachkräfte im Jahr 2018 auf vier Fehltage mehr und waren demnach 18,9 Tage krankgeschrieben. Dabei waren 4,1 Fehltage mit psychischen Erkrankungen begründet, gefolgt von Erkrankungen der Atemwege mit 3,3 Fehltagen. Dieser Trend setzte sich auch im Jahr 2020 fort. So wurden Erzieherinnen und Erzieher zwar am dritthäufigsten aufgrund einer COVID-19-Diagnose krankgeschrieben (nach Pflegepersonal), die meisten Fehlzeiten wurden jedoch weiterhin von psychischen Erkrankungen verursacht, mit einem Anteil von 19,8 % am Gesamtkrankenstand, gefolgt von Muskel-Skelett-Beschwerden (17,9 %) und Krankheiten des Atmungssystems mit 15,3 % [18].

In einer der größten Studien zur Gesundheit von Erzieherinnen und Erziehern (STEGE, 2010–2014) konnten erstmalig empirisch Zusammenhänge zwischen Merkmalen der Strukturqualität und der Wahrnehmung von Belastungen und Ressourcen von pädagogischen Fachkräften (*n* = 2744) gezeigt werden. Deutlich wurden Zusammenhänge zwischen schlechten Arbeitsbedingungen (z. B. unzureichende strukturelle Rahmenbedingungen, Lärm) und psychischen Belastungen [21].

Ziel der AQUA-Studie („Arbeitsplatz und Qualität in Kitas“) war es, Zusammenhänge zwischen den Arbeitsbedingungen und der Arbeitszufriedenheit des Kita-Personals aufzuzeigen. Im Rahmen einer bundesweiten Untersuchung befragten die Autoren [15] über 6600 pädagogische Fachkräfte zu deren Arbeitsbedingungen und dem Ausmaß an Unterstützung seitens der Träger. Auf Grundlage der Ergebnisse erstellte die Arbeitsgruppe einen Index, der die Arbeitsbedingungen in gut, mittel und schlecht einteilt. Es zeigte sich, dass die Arbeitsbedingungen mehrheitlich (71,7 %) eher als schlecht wahrgenommen werden. Die Autoren schlussfolgern, dass schlechte Arbeitsbedingungen u. a. mit niedriger Arbeitszufriedenheit und einer hohen Stressbelastung einhergehen. Fokussiert man die pädagogischen Leitungen, so konnten Schreyer et al. [15] aufzeigen, dass Leitungen, die viel Unterstützung von ihrem Träger erhalten (*n* ges. = 819), sich deutlich seltener belastet fühlen. Wie in der STEGE-Untersuchung [20] sind es auch in der AQUA-Studie die pädagogischen Leitungen, die besonderen Arbeitsbelastungen ausgesetzt sind.

Die Erkenntnisse der BeWAK-Studie zur Wertschätzung und Anerkennung [6] zeigen, dass sich über die Hälfte der befragten Fachkräfte körperlichen und emotionalen Belastungen ausgesetzt sieht und dass knapp 90 % keine entsprechende Honorierung ihrer Leistungen durch die Leitungskräfte empfinden.

Nentwig-Gesemann, Nicolai & Köhler [13] kommen in ihrer Untersuchung zu ähnlichen Ergebnissen bei Leitungskräften. Als weitere Belastungsquelle nennen die Autorinnen ein stetig wachsendes Aufgabenspektrum mit häufig unklaren Anforderungsprofilen, welches zu einer uneindeutigen Verteilung von Zuständigkeiten und Verantwortlichkeiten in den Kindertageseinrichtungen führt.

Das Hauptergebnis der niedersächsischen Untersuchung von Lattner [10] ist, dass Erzieherinnen und Erzieher (*n* = 841) über psychosomatische Beschwerden wie emotionale Erschöpfung berichten, jedoch gleichzeitig angeben, guter psychischer Gesundheit zu sein. Die Ergebnisse der Fragebogenerhebung (*n* = 53) von Losch [12] zeigen auf, dass aus Sicht der Erzieherinnen und Erzieher die Belastung durch Lärm und Stress am Arbeitsplatz den stärksten negativen Einfluss auf ihre Gesundheit hat. Die Analyse von Sica [16] zeigt beispielsweise, dass Situationen mit einem erhöhten Schalldruckpegel (z. B. Essenssituation, Freispiel) von der Mehrheit der Befragten als beanspruchend empfunden werden. Die Autorin verweist zudem auf die besondere Bedeutung der Kombination von emotionalen und sozialen Arbeitsanforderungen.

Die bundesweite GEW-Studie „Wie geht’s im Job?“ bestätigt die bis dato vorliegenden Ergebnisse und erweitert sie um die Erkenntnis, dass sich schlechte Arbeitsbedingungen, niedrige Eingruppierung, mangelnde Aufstiegsmöglichkeiten sowie Personalnotstand und Zeitdruck negativ auf die Arbeitszufriedenheit auswirken [7]. Dennoch geben die Befragten (*n* = 1887) eine relativ hohe allgemeine Arbeitszufriedenheit an.

Backhaus, Hampel & Dadaczynski [2] untersuchten die Auswirkungen arbeitspsychologischer Variablen (Gratifikationskrise und Verausgabungsneigung) auf Depressionen bei pädagogischen Fachkräften (*n* = 1933). Die gemessene Prävalenz für Depressionen lag bei 49,8 %. Die Autoren konnten aufzeigen, dass Gratifikationskrisen und Verausgabungsneigung die Wahrscheinlichkeit von Depressionen signifikant erhöhen.

Auf der Grundlage von zwei Fragebogenerhebungen analysierten Viernickel und Weßels [22] inwiefern Fachkräfte aus Kindertageseinrichtungen (*n* = 1958) und Tagespflegepersonen (*n* = 1721) basierend auf ihren jeweiligen charakteristischen Arbeitsplatzmerkmalen Belastungen wahrnehmen. Insgesamt kommen die Autoren zu dem Schluss, dass beide Berufsgruppen mehr Ressourcen als Belastungen an ihrem Arbeitsplatz wahrnehmen. Das Verhältnis von Ressourcen zu Belastungen fällt jedoch in der Kindertagespflege günstiger aus als in den Tageseinrichtungen. In Kindertageseinrichtungen resultieren die Belastungen v. a. aus finanziellen, räumlichen und personellen Bedingungen, in der Kindertagespflege aus mangelnden Austauschmöglichkeiten und Anerkennungsressourcen.

Die dargestellten Studien zeigen einen Zusammenhang zwischen der Arbeitsbelastung und der gesundheitlichen Situation von Erzieherinnen und Erziehern. Selten werden nur einzelne Faktoren benannt, häufig handelt es sich um Belastungskonstellationen, die gesundheitlich beeinträchtigend wirken. Resümierend lassen sich über die Studienlage hinweg drei Hauptbelastungsfaktoren bei den pädagogischen Fachkräften feststellen:

- *Psychische Belastungen* (Stress und emotionale Erschöpfung u. a. durch Lärm^1^, hohes und komplexes Arbeitspensum, Personalmangel, schlechte strukturelle Rahmenbedingungen);
- *Physische Belastungen* (überwiegend im Muskel-Skelett-Bereich – u. a. durch häufiges Heben, Bücken, ungünstige Sitzpositionen);
- *Infektionskrankheiten* (u. a. häufiges Auftreten von Atemwegserkrankungen).

Neben den Belastungsfaktoren konnten in verschiedenen Untersuchungen jedoch auch zahlreiche Ressourcen ermittelt werden. Diese reichen von sozialen Faktoren wie Unterstützungsleistungen durch Kolleginnen und Kollegen und Vorgesetzten bis hin zu organisationalen Ressourcen wie großen Entscheidungsspielräumen, Unterstützungsleistungen durch den Träger sowie Fort- und Weiterbildungsmöglichkeiten. Als weiterer wichtiger Schutzfaktor in der alltäglichen pädagogischen Arbeit wurden die hohe Identifikation mit dem Beruf als auch die Anerkennung von außen genannt.

Aufbauend auf den skizzierten Erkenntnissen geht vorliegende Analyse einen Schritt weiter und vergleicht die psychische und emotionale Erschöpfung bei Erziehern und Erzieherinnen mit Angehörigen anderer Berufsgruppen.

## Methodisches Vorgehen und Ergebnisse

Analysiert wird die vom Bundesinstitut für Berufsbildung (BIBB) und der Bundesanstalt für Arbeitsschutz und Arbeitsmedizin (BAuA) durchgeführte „BIBB/BAuA-Erwerbstätigenbefragungen – Arbeit und Beruf im Wandel, Erwerb und Verwertung beruflicher Qualifikationen“ von 2006, 2012 und 2018. Tab. 2 zeigt die Fallzahlen und die Altersverteilungen.

**Table 2:** 

Jahr	2006				2012				2018			
	Erzieherinnen/Erzieher		Andere Berufe		Erzieherinnen/Erzieher		Andere Berufe		Erzieherinnen/Erzieher		Andere Berufe	
Altersgruppe	*n*	%	*n*	%	*n*	%	*n*	%	*n*	%	*n*	%
15–31	45	16,7	4046	20,7	78	24,0	3964	20,4	102	24,8	3831	19,9
32–41	81	30,4	5677	29,0	67	20,8	4422	22,8	98	23,7	4253	22,1
42–54	124	46,3	7890	40,3	150	46,3	8233	42,5	135	32,8	7445	38,7
55–67	18	6,6	1978	10,1	29	9,0	2773	14,3	72	17,5	3696	19,2
Gesamt	268	–	19.591	–	324	–	19.392	–	407	–	19.225	–
Männlich	21	7,7	11.234	56,9	26	8,1	11.001	55,8	55	13,4	10.887	55,5
Weiblich	248	92,3	8497	43,1	299	91,9	8710	44,2	357	86,6	8713	44,5

Psychische Erschöpfung wird anhand eines Indexes aus sechs möglichen Symptomen ermittelt und für die Berufsgruppen für die Jahre 2006, 2012 und 2018 dargestellt (Mittelwerte, Konfidenzintervalle). Dies auch für die emotionale Erschöpfung (nur ein Item) für die Jahre 2012 und 2018. Die Kategorisierung der Erzieherinnen und Erzieher erfolgt über die Zuteilung zu unterdurchschnittlicher psychischer Erschöpfung (0–2 Symptome) bzw. überdurchschnittlicher Erschöpfung (3–6 Symptome). Emotionale Erschöpfung resultiert aus einer binären Variable (in den letzten 12 Monaten während der Arbeit bzw. an Arbeitstagen häufig aufgetreten oder nicht). Gruppe 1 (besonders schwach erschöpft) und Gruppe 4 (besonders stark erschöpft) sind die größten Gruppen, da beide Erschöpfungsformen tendenziell zusammenhängen.

Belastungs- und Resilienzfaktoren in beiden Berufsgruppen werden für das Jahr 2018 dargestellt (Mittelwerte und Konfidenzintervalle). Dazu gehören (1) vier Items zur qualitativen und quantitativen Überforderung, (2) drei Items zu Belastungsfaktoren, die v. a. bei Erzieherinnen und Erziehern stark ausgeprägt sind (Lärm, störende Geräusch, Mikroorganismen), (3) fünf Items zur sozialen Unterstützung am Arbeitsplatz (Gemeinschaftsgefühl, Hilfe/Unterstützung durch Kolleginnen und Kollegen, Zusammenarbeit mit diesen, Hilfe/Unterstützung durch Vorgesetzte, Lob von diesen) sowie (4) die Möglichkeit, nach der Arbeit abschalten zu können. Da es sich weitgehend um Fragen mit nichtmetrischen Antwortskalierungen handelt (Antwortmöglichkeiten „nie“, „selten“, „manchmal“, „häufig“), erfolgt die Indexbildung über eine Dichotomisierung (Faktor tritt häufig auf oder nicht); im Anschluss wird die Anzahl der häufigen Faktoren jeweils addiert. Zu (2) wird die Folgefrage, ob dies, falls vorliegend, als belastend empfunden wird, verwendet.

Aufgrund des gemeinsamen Auftretens verschiedener Faktoren werden mehrere Faktoren zusammengefasst. Hierdurch liegt keine Multikollinearitätsproblematik vor, die Toleranzwerte liegen über 0,8. Die logistische Regression besteht somit aus sieben unabhängigen Variablen (quantitative Überforderung, qualitative Überforderung, Lärm/Geräusche, Mikroorganismen, Unterstützung Kolleginnen und Kollegen, Unterstützung Vorgesetzte, Abschalten nach der Arbeit). Die logistische Regression kann die Wahrscheinlichkeit der Zugehörigkeit zur besonders stark belasteten Gruppe gegen die Gegenwahrscheinlichkeit der Zugehörigkeit zur besonders stark belasteten Gruppe mit einer erklärten Varianz (Nagelkerkes Pseudo-R-Quadrat) von 41 % gut erklären.

### Entwicklung und Ausmaß psychischer Erschöpfung

**Figure 1:**
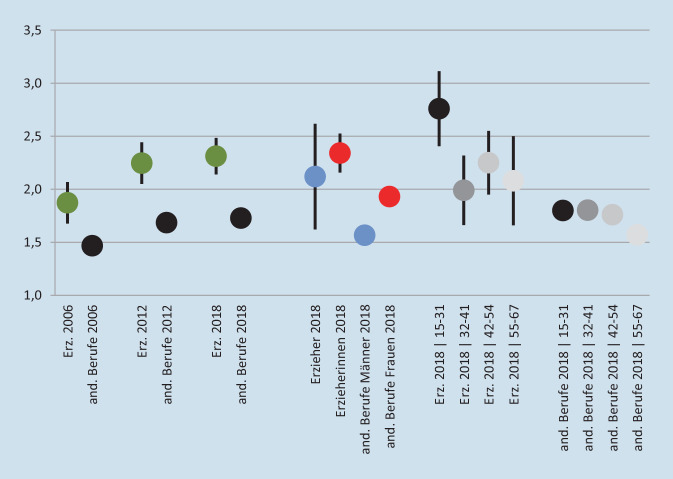

In Abb. 1 wird gezeigt, dass die psychische Erschöpfung (Index aus den sechs Symptomen Kopfschmerzen; nächtliche Schlafstörungen; allgemeine Müdigkeit, Mattigkeit oder Erschöpfung; Magen- oder Verdauungsbeschwerden; Nervosität oder Reizbarkeit; Niedergeschlagenheit) bereits im Jahr 2006 höher war als bei den anderen Berufen (1,87 zu 1,47). Dieser Abstand hat sich v. a. durch den überdurchschnittlich starken Anstieg bis 2012 noch vergrößert (2,25 zu 1,69). Das Muster setzt sich mit einem abgeschwächten Anstieg bis 2018 fort (2,31 zu 1,73). Abb. 1 zeigt auch (für 2018) die Verteilung der psychischen Erschöpfung innerhalb beider Gruppen. Zunächst zeigt sich eine etwas geringere psychische Erschöpfung bei den Erziehern (2,12) als den Erzieherinnen (2,34). Der Unterschied ist statistisch nicht signifikant und mit 0,22 Skalenpunkten wesentlich geringer als der Geschlechterunterschied bei den anderen Berufen. Dies ist plausibel, da die Unterschiede in den anderen Berufen auch durch geschlechterspezifische Über- und Unterrepräsentationen in besonders belasteten Berufsgruppen zustande kommen, was innerhalb des erzieherischen Berufs nicht möglich ist. Im Vergleich der Altersgruppen bemerkenswert sind die hohen Werte bei der jüngsten Gruppe (15–31 Jahre). Der Vergleich mit den anderen Berufen zeigt, dass dies nicht einem gewöhnlichen Muster über viele Berufsgruppen hinweg entspricht, sondern ein Problem bei Erzieherinnen und Erziehern und ggf. einzelnen anderen Berufen ist. Die Altersgruppe ist aus zwei Gründen hochrelevant: Erstens ist in dieser Lebensphase die Wahrscheinlichkeit einer beruflichen Neuorientierung noch höher als in späteren Lebensphasen. Und zweitens können berufliche Belastungen in dieser Lebensphase darüber entscheiden, ob, wann und in welchem Umfang nach einer beruflichen Pause aufgrund von Familiengründungen in den Beruf zurückgekehrt wird. Dies betrifft den erzieherischen Beruf besonders stark, da in dieser Berufsgruppe Frauen stark überrepräsentiert sind und die innerpartnerschaftliche Aufgabenverteilung nach der Familiengründung noch immer weitgehend traditionellen Mustern folgt (z. B. nur kurze familienbedingte berufliche Auszeit über die derzeit (2022) zwei *Vätermonate* im Elterngeld). Eine genauere Analyse der jüngsten Altersgruppe zeigt, dass zwar die wenigen Erzieher (*n* = 12) durch ihren hohen Mittelwert (3,77) den Wert der Altersgruppe etwas nach oben verschieben, jedoch auch bei den Erzieherinnen (Mittelwert 2,62, *n* = 90) der Wert im Vergleich zu den anderen Altersgruppen hoch ist.

### Entwicklung und Ausmaß emotionaler Erschöpfung

**Figure 2:**
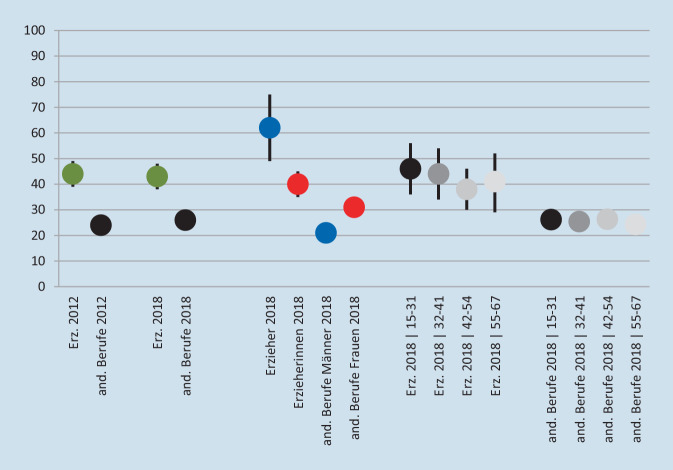

Seit 2012 wird direkt nach häufiger „emotionaler Erschöpfung“ gefragt (Antwort binär: ja/nein). Abb. 2 zeigt im Vergleich zwischen Erzieherinnen und Erziehern und den Angehörigen anderer Berufe ein ähnliches Bild wie Abb. 1 zur psychischen Erschöpfung. Im Geschlechtervergleich fällt auf, dass Erzieherinnen signifikant seltener (zu ca. 40 %) eine emotionale Erschöpfung angeben als Erzieher (ca. 62 %). Aufgrund von Abb. 1 wäre ein umgekehrtes Ergebnis zu erwarten gewesen. Das Ergebnis für die anderen Berufe ähnelt jenem für die psychische Erschöpfung. Im Vergleich der Altersgruppen zeigt zwar auch bei den Erzieherinnen und Erziehern die jüngste Gruppe den höchsten Wert (ca. 46 %); dieser ist allerdings nur leicht höher als bei den anderen Gruppen (44 %, 37 % und 41 %). Bei den andern Berufen hat die Gruppe 42–54 die höchsten, die Gruppe 55–67 die niedrigsten Werte.

Der Zusammenhang zwischen dem Index zur psychischen Erschöpfung und der Angabe zur emotionalen Erschöpfung zeigt sich im Vergleich zwischen jenen pädagogischen Fachkräften, die keine emotionale Erschöpfung angeben und jenen, die diese angeben. Die erste Gruppe berichtet im Mittelwert über nur 1,54 Symptome psychischer Erschöpfung (95 % KI 1,35–1,73; *n* = 235), die zweite Gruppe über 3,35 Symptome (95 % KI 3,10–3,59; *n* = 176). Dennoch muss unterschieden werden: Psychische Erschöpfung bezieht sich teilweise auf körpernahe Symptome (z. B. Kopfschmerzen, Schlafstörungen, Magen- oder Verdauungsbeschwerden), während die emotionale Erschöpfung eher körperferne Eigenschaften beinhaltet (z. B. Gefühl von Sinnlosigkeit oder mangelnder Anerkennung).

### Psychische und emotionale Erschöpfung: besonders belastete Erzieherinnen und Erzieher

**Figure 3:**
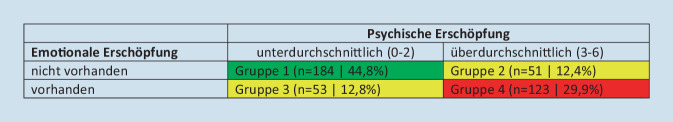

In Abb. 1 wird deutlich, dass Erzieherinnen stärker unter psychischer Erschöpfung leiden als Erzieher, während Abb. 2 darlegt, dass Erzieher stärker unter emotionaler Erschöpfung leiden als Erzieherinnen. Und dies, obwohl beide Beschwerden deutlich zusammenhängen, wie der vorangegangene Absatz zeigt. Im Folgenden wird eine Variable für die psychisch-emotionale Gesamterschöpfung anhand beider Dimensionen konstruiert. Dies erlaubt die Identifizierung besonders stark oder schwach belasteter Gruppen. Hier werden die Erzieherinnen und Erzieher gemäß der Darstellung in Abb. 3 kategorisiert. Der Mittelwert für die psychische Erschöpfung liegt bei 2,31 (Abb. 1). Erzieherinnen und Erzieher mit 0 bis 2 Symptomen gelten als unterdurchschnittlich mit psychischer Erschöpfung belastet (*n* = 237), ab 3 Symptomen als überdurchschnittlich belastet (*n* = 174). Eine emotionale Erschöpfung liegt bei 235 Personen nicht vor, bei 176 Personen liegt diese vor. Überkreuzt ergibt sich ein Bild wie in Abb. 3 dargestellt.

Die Gruppen 1 (schwach belastet) und 4 (stark belastet) sind erwartungsgemäß stark besetzt. Wie Abb. 2 bereits nahelegt, ist bei den pädagogischen Fachkräften die Gruppe 4 mit 29,9 % wesentlich stärker besetzt als bei den anderen Berufen (18,0 %), während die Gruppe 1 schwächer besetzt ist (44,8 % zu 61,8 %). Die Gruppen 2 und 3 (jeweils mittelstark belastet) sind demnach eher schwach besetzt. Erwartungsgemäß nach Abb. 1 und 2 besteht die Gruppe 2 in Abb. 3 sehr deutlich aus Frauen (95,7 %; *n* = 49) und beinhaltet kaum Männer (4,3 %; *n* = 2), während Gruppe 3 einen deutlich höheren Männeranteil hat (29,6 %; *n* = 16; Frauenanteil 70,4 %; *n* = 37); dies vor dem Hintergrund eines allgemeinen Männeranteils von 13,4 % (*n* = 55). Das mittlere Alter ist bei den Gruppen 2 bis 4 mit 41–42 Jahren nahezu identisch, Gruppe 1 ist mit 43 Jahren etwas älter.

### Belastungs- und Resilienzfaktoren

In diesem Abschnitt wird die Frage beantwortet, was die Zugehörigkeit zur besonders stark belasteten Gruppe 4 im Gegensatz zur Zugehörigkeit zur besonders schwach belasteten Gruppe 1 begünstigt. Als mögliche Determinanten können gelten:

#### (1a) *Stressoren quantitativer Überforderung.*

- Beschäftigte stehen unter starkem Termin- oder Leistungsdruck.
- Beschäftigte müssen bis an die Grenzen ihrer Leistungsfähigkeit gehen.

#### (1b) *Stressoren qualitativer Überforderung.*

- Es werden Dinge verlangt, die nicht gelernt wurden oder die nicht beherrscht werden.
- Die Tätigkeit bringt in Situationen, die gefühlsmäßig belastend sind.

Da aufgrund der ordinalen Antwortskalierung in den vier Fragen zur qualitativen und quantitativen Überforderung keine Mittelwerte berechnet werden können, wird für jede Person die Anzahl an Stressoren gezählt, die „häufig“ vorkommen.

#### (2).

Die Wahrnehmung verschiedener anderer beruflicher Belastungen wird zweistufig abgefragt: Zunächst mit der Frage, wie häufig eine potenzielle Belastungssituation vorkommt („nie“, „selten“, „manchmal“, „häufig“) und für diejenigen Befragten, die „häufig“ angeben, die Folgefrage, ob dies belastend sei (nein, ja). Einige mögliche Belastungen kommen unter Erzieherinnen und Erziehern sehr selten vor, wie z. B. blendendes Licht. Andere mögliche Belastungen sind stark auf körperliche Beschwerden bezogen (z. B. im Stehen arbeiten, lang im Sitzen arbeiten). Ausgewählt wurden drei Faktoren, denen sich Erzieher und Erziehrinnen überdurchschnittlich stark und häufig ausgesetzt fühlen und die gleichzeitig von vielen als belastend erlebt werden. Dies sind die Belastungen durch …

- Lärm,
- störende Geräusche,
- Mikroorganismen wie Krankheitserreger, Bakterien, Schimmelpilze oder Viren.

**Figure 4:**
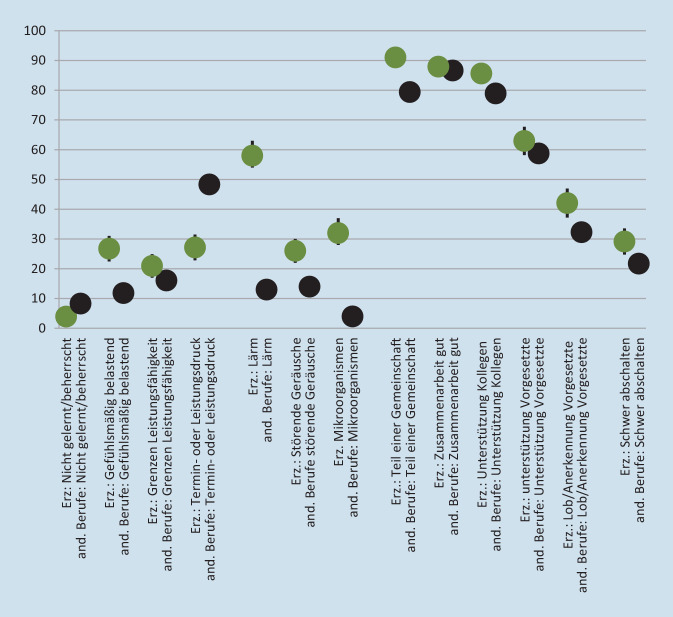

Insgesamt 97,2 % der Erzieherinnen und Erzieher, die häufig störende Geräusche wahrnehmen und als belastend empfinden, nehmen auch häufig Lärm wahr, den sie als belastend empfinden. Da wesentlich häufiger Lärm wahrgenommen wird als störende Geräusche, liegt der Anteil umgekehrt bei 43,6 %. Beide Variablen werden in einen Index von 0 (weder belastender Lärm noch belastende störende Geräusche) über 1 (ein Faktor vorhanden) bis 2 (beide Faktoren vorhanden) zusammengefasst (Abb. 4).

#### (3).

Die Variable „Unterstützung am Arbeitsplatz“ wird aus fünf Items gebildet:

- Wie häufig kommt es vor, dass Sie sich an Ihrem Arbeitsplatz als Teil einer Gemeinschaft fühlen?
- Wie oft empfinden Sie die Zusammenarbeit zwischen Ihnen und Ihren Arbeitskollegen/Mitarbeitern als gut?
- Wie oft bekommen Sie Hilfe und Unterstützung für Ihre Arbeit von Kollegen, wenn Sie diese brauchen?
- Und wie oft bekommen Sie Hilfe und Unterstützung für Ihre Arbeit von Ihrem direkten Vorgesetzten, wenn Sie diese brauchen?
- Wie oft gibt Ihnen Ihr direkter Vorgesetzter Lob und Anerkennung, wenn Sie gute Arbeit leisten?

Aufgrund der nichtmetrischen Antwortskalierung („nie“, „selten“, „manchmal“, „häufig“) musste dichotomisiert werden in „häufig“ und „nicht häufig“. Zwar wird auch hier die Folgefrage gestellt, ob das als belastend empfunden wird (wenn das „nie“ der Fall ist). Diese Variablen sind jedoch nicht verwendbar, da nur ein sehr geringer Anteil an pädagogischen Fachkräften bei diesen Fragen „nie“ angibt (0,3 %, 0,4 %, 0,2 %, 1,6 %, 5,1 %).

#### (4).

Das Abschalten nach der Arbeit wurde ebenfalls mit einer nichtmetrischen Antwortskala („nie“, „selten“, „manchmal“, „häufig“) abgefragt. Hier gibt es keine Folgefrage nach der Belastung für diejenigen, denen das schwerfällt.

Seltener als von den Angehörigen anderer Berufe werden von Erzieherinnen und Erziehern Dinge verlangt, die sie nicht gelernt haben oder die sie nicht beherrschen. In gefühlsmäßig belastende Situationen kommen sie allerdings zu weitaus höheren Anteilen „häufig“ (26,7 % zu 11,9 %). Auch an die Grenzen ihrer Leistungsfähigkeit kommen sie zu höheren Anteilen häufig, allerdings mit deutlich weniger Termin- oder Leistungsdruck. Auffällig ist der hohe Anteil an pädagogischen Fachkräften, die sich häufig Lärm ausgesetzt fühlen und dies als belastend empfinden. Mit 58,5 % zu 12,7 % ist die Wahrscheinlichkeit dieser Belastung ca. 4,6-mal so hoch wie für Angehörige anderer Berufe. Bei den störenden Geräuschen ist der Unterschied geringer. Bei den Mikroorganismen, das heißt, vor allem Krankheitserregern, ist mit 32,2 % zu 4,0 % die Wahrscheinlichkeit für Erzieherinnen und Erziehern etwa 8‑mal so hoch. Bereits vor der Corona-Pandemie wurden hier von der Berufsgruppe deutliche Probleme gesehen. Unter allen ca. 350 Berufen nehmen Erzieherinnen und Erziehern hier den zweithöchsten Wert ein. Dieser liegt zwar unter jenem der Kinderpfleger (ca. 35 %), die im gleichen Arbeitsfeld tätig sind, aber oberhalb sogar des Personals im Gesundheitswesen (Krankenpfleger und Hebammen ca. 28 %, Ärzte ca. 17 %).

Allgemein werden das Gemeinschaftsgefühl sowie die kollegiale Zusammenarbeit und Unterstützung von einem hohen Anteil der Erwerbstätigen als „häufig“ vorhanden oder gut empfunden. Erzieherinnen und Erzieher liegen hier, v. a. beim Gemeinschaftsgefühl, noch oberhalb des bereits hohen Werts für die anderen Berufe. Niedriger fallen die Werte für die Vorgesetzten aus, was auch an den selteneren Gelegenheiten liegen kann. Aber hier sind die Angaben ebenfalls positiver als in den anderen Berufen. Die drei Fragen zu Gemeinschaftsgefühl am Arbeitsplatz, Zusammenarbeit mit Kolleginnen und Kollegen und kollegiale Unterstützung müssen in einen Index zusammengeführt werden (0- bis 3‑mal „häufig“ genannt), da die Angabe „häufig“ stark zusammenhängt. So geben 96,6 % derer, die häufig eine gute Zusammenarbeit sehen auch an, sich „häufig“ als Teil einer Gemeinschaft zu sehen (umgekehrt 93,2 %), und 95,0 % derer, die häufig Hilfe und Unterstützung durch Kolleginnen und Kollegen bekommen, auch häufig ein starkes Gemeinschaftsgefühl an (umgekehrt 89,3 %; die umgekehrt niedrigeren Werte ergeben sich daraus, dass die Variable „Gemeinschaftsgefühl“ allgemein etwas höhere Zustimmung hat). Für die beiden Variablen zu den Kolleginnen und Kollegen liegen die Werte auch jeweils über 90 %. Die Zusammenhänge zu den Vorgesetzen sind zumindest in eine Richtung naturgemäß schwächer, da diese Variablen weniger Zustimmung erfahren (z. B. wer die Unterstützung oder das Lob durch Vorgesetzte „häufig“ sieht, fühlt sich meist auch „häufig“ als Teil einer Gemeinschaft, aber umgekehrt wesentlich seltener). Die beiden Variablen zu den Vorgesetzten wurden auch in einen Index (0–2) zusammengefasst (91,7 % derer, die häufig Lob und Anerkennung erfahren, berichten auch über häufige Unterstützung. Da Lob und Anerkennung durch Vorgesetzte seltener sind als Unterstützung, liegt der umgekehrte Wert bei 61,4 %). Für Erzieherinnen und Erzieher ist die Wahrscheinlichkeit, dass es häufig schwerfällt, nach der Arbeit abzuschalten, im Vergleich zu den Angehörigen anderer Berufe um etwa ein Drittel erhöht.

### Determinanten psychischer und emotionaler Erschöpfung

**Figure 5:**
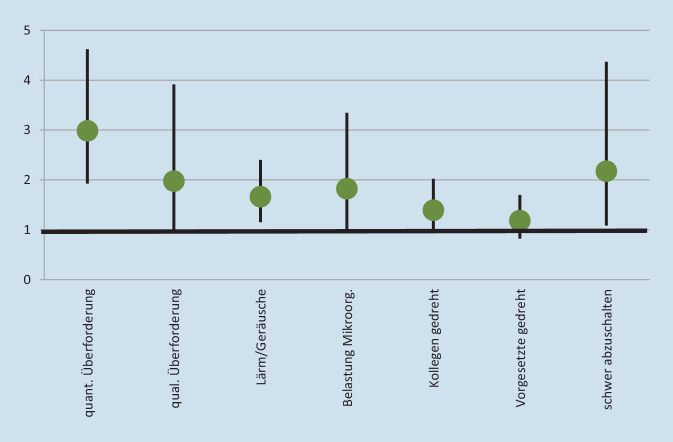

In Abb. 5 wird veranschaulicht, wie die verschiedenen Faktoren die Wahrscheinlichkeit beeinflussen, zur psychisch und emotional erschöpften Gruppe 4 anstatt zur weder psychisch noch emotional erschöpften Gruppe 1 zu gehören. Die Wahrscheinlichkeitsveränderung pro Schritt auf den unabhängigen Variablen ergibt sich aus Exp(B) minus 1. Der Punkt 1 bedeutet demnach, dass es keinen Einfluss gibt, unter 1 ist der Einfluss negativ, über 1 ist der Einfluss positiv. Jedoch kann die Wahrscheinlichkeitsveränderung bei mehrstufigen unabhängigen Variablen nicht linear interpretiert werden, wodurch sich eine Beschränkung auf die Richtung der Effekte und auf signifikante und insignifikante Effekte empfiehlt.

Deutlich wird die Wahrscheinlichkeitsveränderung der Gruppenzugehörigkeit v. a. bei der quantitativen Überforderung. Diese Überforderung ist mit ca. 21 % (Grenzen der Leistungsfähigkeit) und ca. 27 % (Termin- oder Leistungsdruck) der Erzieherinnen und Erzieher, die diese Faktoren als „häufig“ bezeichnen, wesentlich weiter verbreitet als die Notwendigkeit, Dinge zu tun, die nicht gelernt wurden oder nicht beherrscht werden. Und es sind, das zeigen auch Analysen mit jeweils einzelnen Faktoren der Überforderung, ihre Bestandteile jeweils relevanter für die psychische und emotionale Erschöpfung als die gefühlsmäßige Belastung. Das bedeutet, diese Belastungsfaktoren sind bei pädagogischen Fachkräften stark ausgeprägt und sie begünstigen auch deutlich die Erschöpfung. Auch wenn *p*-Werte aufgrund der geringen Fallzahlen erhöht werden, d. h. die Wahrscheinlichkeit signifikanter Zusammenhänge durch längere Konfidenzintervalle sinkt, zeigt sich dies auch in dem hohen Signifikanzniveau der quantitativen Überforderung und dem knapp insignifikanten Einfluss der qualitativen Überforderung. Innerhalb dieser ist die gefühlsmäßige Belastung, als Einzelvariable aufgenommen, zwar mit Exp (B) von 1,912 schwächer als die einzelne aufgenommen der Termin- und Leistungsdruck. Exp(B) 3,184***, oder das Arbeiten bis an die Grenze der Leistungsfähigkeit, Exp(B) 2,239**, jedoch auf dem 5 %-Niveau statistisch noch signifikant. Kurz gesagt: Zu hohe Arbeitsmenge ist ein bedeutsamer Belastungsfaktor. Gefühlsmäßig belastende Situationen sind für die psychische und emotionale Erschöpfung weniger relevant. Ein subjektiver Mangel an fachlicher Kompetenz kommt sehr selten vor und spielt (auch als schwache und insignifikante Einzelvariable) kaum eine Rolle für die psychische und emotionale Erschöpfung.

Lärm und störende Geräusche, ebenfalls ein Index aus drei Skalenpunkten (0 bis 2) und demnach mit zwei Schritten, sind nicht nur aufgrund der gezeigten Einflüsse auf die psychische und emotionale Erschöpfung relevant, sondern auch aufgrund ihrer sehr hohen Verbreitung als Belastungsfaktor unter Erzieherinnen und Erziehern. Dies gilt auch für den Umgang mit Mikroorganismen (Abb. 4).

Das kollegiale Verhältnis ist bei der erzieherischen Berufsgruppe sehr gut und für die psychische und emotionale Erschöpfung kaum bedeutsam. Dies gilt ähnlich auch für das Verhalten von Vorgesetzten. Es ist zwar noch Spielraum nach oben (Abb. 4), jedoch könnte dies eher die allgemeine Arbeitszufriedenheit steigern und weniger die Erschöpfung reduzieren (Abb. 5).

Letztlich kann gezeigt werden, dass die Wahrscheinlichkeit der Zugehörigkeit zur besonders stark belasteten Gruppe im Gegensatz zur Zugehörigkeit zur besonders schwach belasteten Gruppe durch das häufige schwere Abschalten können nach der Arbeit signifikant erhöht wird (Abb. 5). Abb. 4 zeigt, dass Erzieherinnen und Erzieher hiervon stärker betroffen sind als die Angehörigen anderer Berufe. Die erklärte Varianz des Modells ist mit ca. 41 % als vergleichsweise hoch einzustufen. Es liegt mit Toleranzwerten von durchgehend über 0,8 keine Multikollinearitätsproblematik vor.

## Diskussion und Fazit

**Figure 6:**
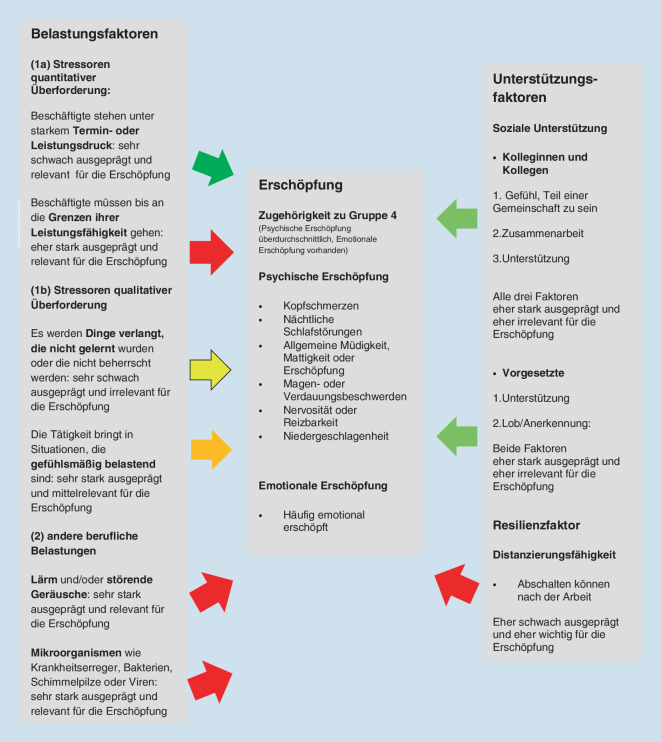

Die vorliegenden Ergebnisse haben einen ersten Blick auf die Belastungen von Erzieherinnen und Erziehern für den Zeitraum 2006 bis 2018 ermöglicht. Deutlich wird die hohe psychische und emotionale Erschöpfung, die in den Jahren 2012 bis 2018 weitgehend stabil blieb (Abb. 1 und 2). Deutlich wird auch die stärkere Konfrontation von Erzieherinnen und Erziehern mit widrigen Umständen, v. a. gefühlsmäßig belastenden Situationen, Lärm und der Umgang mit Mikroorganismen wie Krankheitserregern, Bakterien, Schimmelpilzen oder Viren. Zudem können pädagogische Fachkräfte nach der Arbeit vergleichsweise schwer abschalten (Abb. 4). Relativ viele Erzieherinnen und Erzieher sind deutlich psychisch und emotional erschöpft. Es wurde ermittelt, welche Faktoren dazu führen, dass Personen innerhalb der Berufsgruppe überdurchschnittlich stark psychisch erschöpft sind und zudem eine emotionale Erschöpfung angeben (was für ca. 30%der Gruppe zutrifft) anstatt das Gegenteil anzugeben (was für ca. 45%der Gruppe zutrifft). Abb. 6 kombiniert, (a) inwiefern die Belastungsfaktoren, Unterstützungsfaktoren sowie der Resilienzfaktor bei den Erzieherinnen und Erziehern im Vergleich zu den Angehörigen aller anderen Berufsgruppen besonders stark oder schwach ausgeprägt sind mit (b) der Relevanz dieser Faktoren für die Erschöpfung. Die fünfstufige Farbgebung fasst die empirischen Ergebnisse zusammen und zeigt auf der negativen Seite (orange und v. a. rot), welche Ansatzpunkte vielversprechend für eine Reduzierung der Erschöpfung sind. Anders gesagt: Hier besteht Potenzial für Verbesserungen der Faktoren (durch die vergleichsweise schlechten Werte bei Erzieherinnen und Erziehern), und diese Faktoren beeinflussen die Erschöpfung.

Deutlich wird, dass die quantitative Überforderung bei Erzieherinnen und Erziehern mittelhoch ist (im Vergleich zu anderen Berufen eher das Arbeiten bis an die Grenzen der Leistungsfähigkeit als Termin- oder Leistungsdruck) und dass gerade dieser Faktor die Zugehörigkeit zur erschöpftesten Gruppe wahrscheinlicher macht.

Zur Reduktion von psychischen und emotionalen Belastungsfaktoren für Erzieherinnen und Erzieher und damit verbundenen Folgeproblemen bieten die Ergebnisse konkrete Ansatzpunkte. Bedeutsamer als der allgemeine Befund, dass pädagogisches Personal insgesamt ein vergleichsweise hohes Belastungserleben aufweist, ist der Bezug zu konkreten Einzelfaktoren, die erstens stark ausgeprägt und zweitens bedeutsam für die psychische und emotionale Erschöpfung sind. Um diesem gesamten skizzierten Spektrum an Belastungsfaktoren im Sinne der Strukturqualität zu begegnen, müssen neben einrichtungsspezifischen Voraussetzungen auch politisch verantwortete Rahmenbedingungen angestoßen werden. Es besteht Handlungsspielraum durch eine bessere Personalausstattung oder eine Reduzierung der Aufgabenbereiche. Gefühlsmäßig belastende Situationen sind für die Erschöpfung weniger relevant und auch schwerer vermeidbar (Abb. 6). Lärm und störende Geräusche kommen sehr häufig vor und sind relevant für die Erschöpfung. Allerdings liegt nahe, dass praktische Verbesserungen wesentlich schwerer zu entwickeln sind als bei der quantitativen Überforderung. Thinschmidt [20] schlägt vor, neben bautechnischen Schritten auch organisatorische und pädagogische Maßnahmen (z. B. Entzerrung von Stoßzeiten und eine Verteilung von Freispielphasen) zur Reduktion des Lärms in Erwägung zu ziehen. Die Aussagen für Lärm und Geräusche gelten auch für die Belastung durch Mikroorganismen, wobei an dieser Stelle eine praktische Verbesserung für die sehr stark belastete Gruppe darin liegen könnte, auch außerhalb von Pandemien wesentlich stärker darauf zu achten, dass Kinder mit Krankheitssymptomen den Kindertageseinrichtungen fernbleiben. Dies kann im Gegensatz zu einer Behebung des Fachkräftemangels schneller umgesetzt werden, verlagert allerdings das Problem hin zu den Familien bzw. Trägern. Das kollegiale Verhältnis ist sehr gut und für die Erschöpfung kaum relevant, sodass kaum Verbesserungen möglich sind. Erzieherinnen und Erzieher können vergleichsweise schwer nach der Arbeit abschalten. Allerdings liegt nahe, dass es sich hierbei weniger um unabhängig bestehenden Faktor handelt, sondern dass Bedingungen innerhalb der Arbeitszeit das schwere Abschalten fördern. Demnach könnten Verbesserungen z. B. in Bezug auf die Arbeitsmenge gleichzeitig diesen Faktor abschwächen. Die eigenen Ergebnisse decken sich mit der OECD-Fachkräftebefragung aus dem Jahr 2018. Im internationalen Vergleich fühlt sich das in Deutschland tätige pädagogische Personal erheblichem Stress ausgesetzt und erfährt nur wenig Wertschätzung. Länderübergreifend werden fehlende finanzielle, strukturelle und personale Ressourcen genannt. Zusätzliche Aufgaben, die aufgrund von Personalausfällen übernommen werden müssen, werden vor allem in Deutschland als besonders psychisch belastend erlebt [3].

Auch wenn einzelne Studien erkennen lassen [7, 22], dass ein Teil der Fachkräfte mit ihren Arbeitsbedingungen in gewissen Bereichen zufrieden ist, zeigen die Entwicklung, das Ausmaß und die Determinanten von Belastungsfaktoren, dass Verbesserungen nötig und möglich sind.

## Footnotes

1 Einzelne Belastungen werden in den Untersuchungen separat aufgeführt – z. B. Lärm nicht als Unterkategorie psychischer Belastungen, sondern als eigene Belastungsquelle [2, 14].
